# Enhancing Tennis Practice: Sensor Fusion and Pose Estimation with a Smart Tennis Ball

**DOI:** 10.3390/s24165306

**Published:** 2024-08-16

**Authors:** Yu Kit Foo, Xi Li, Rami Ghannam

**Affiliations:** James Watt School of Engineering, University of Glasgow, Glasgow G12 8QQ, UK; fooyukit@outlook.com (Y.K.F.); 2756830l@student.gla.ac.uk (X.L.)

**Keywords:** wearable sensors, tennis, haptic feedback

## Abstract

This article demonstrates the integration of sensor fusion for pose estimation and data collection in tennis balls, aiming to create a smaller, less intrusive form factor for use in progressive learning during tennis practice. The study outlines the design and implementation of the Bosch BNO055 smart sensor, which features built-in managed sensor fusion capabilities. The article also discusses deriving additional data using various mathematical and simulation methods to present relevant orientation information from the sensor in Unity. Embedded within a Vermont practice foam tennis ball, the final prototype product communicates with Unity on a laptop via Bluetooth. The Unity interface effectively visualizes the ball’s rotation, the resultant acceleration direction, rotations per minute (RPM), and the orientation relative to gravity. The system successfully demonstrates accurate RPM measurement, provides real-time visualization of ball spin and offers a pathway for innovative applications in tennis training technology.

## 1. Introduction

Today, sensors are compact, fast, and capable of wireless communication with other devices, revolutionizing their use across various fields, including sports [1,2,3]. For instance, during the 2022 FIFA World Cup in Qatar, the FIFA committee introduced a soccer ball equipped with a local positioning system (LPS) and inertial measurement units (IMU). These sensors enabled the tracking of data such as speed, spin rate, and distance traveled, enhancing the precision in determining the kick point for offside calls and providing teams with valuable performance analytics. This innovation is part of a broader trend where sensor technology, such as the Adidas GMR (pronounced “Gamer”), which previously monitored metrics like passes, shot power, and pace, is increasingly incorporated into sports training as wearable devices [4,5,6]. This allows for the quantification and tracking of an athlete’s performance, facilitating targeted improvements during training [7].

In tennis, stroke depends on multiple factors. Notably, the initial spin applied by the opposing player is based on the motion of the racquet, the speed of approach of the ball, and the direction of the ball [8,9]. The player needs to react accordingly and apply just enough power in the return stroke to keep it in bounds while those factors are in place. The ball spin after returning the ball matters since it applies a Magnus effect on the ball. The spin of the ball creates a difference in the pressure of fluids due to slow and fast airflow in the opposite direction of the ball, resulting in a force pushing the ball to the direction of lower pressure [10]. A simple application of the Magnus effect in tennis is the use of topspin to cause a downward swerve to make a more powerful shot stay inbound. Since there would be an initial spin from the opposing player’s stroke, the resultant spin is the factor that determines the Magnus effect force.

In this article, we demonstrate a sensor-based system that gives tennis players quantitative feedback in the form of rotation per minute (RPM), which is produced by the player’s stroke and the plane of rotation of the ball. Using this technology, a tennis player can progressively work on their return stroke with quantitative feedback on how well they are doing.

## 2. Literature Review

### 2.1. Similar Technologies Integrating Sensors in a Ball

Learning to play a sport requires consistent practice and expert guidance. According to Reid et al. [11], there are two types of feedback that athletes can use to monitor their progress and development: “intrinsic” and “augmented” feedback. Intrinsic feedback is derived from the sensory information available to the player during the execution of a shot, such as the sound of the shot or their balance afterwards. On the other hand, augmented feedback involves the use of equipment or technology to provide additional data, such as ball speed and spin, which can help the player enhance their performance.

In recent years, the integration of sensors into sports equipment, like balls, has become a prominent trend. This approach serves as a means of providing augmented feedback to players, facilitating further improvement in their skills. For example, the introduction of the Adidas miCoach smart ball in 2014 marked a significant milestone in soccer training and research [12]. By connecting to users’ smartphones via Bluetooth, the miCoach smart ball provided valuable data such as velocity, RPM (revolutions per minute), and kick rating, enabling players and researchers to delve deeper into the intricacies of techniques like the knuckleball [13]. This innovative technology not only facilitated more precise data collection but also played a crucial role in assessing the optimal spin rate and velocity for executing successful knuckleballs, a technique renowned for its unpredictability.

The integration of sensors in sports balls has seen diverse applications across multiple sports disciplines, not just in soccer. This technological advancement has provided significant benefits in terms of performance analysis, training enhancement, and even fan engagement. Here are some notable commercial examples:

- Basketball: 94Fifty Smart Sensor Basketball: This basketball, embedded with sensors, measures the force and spin with which it is thrown and provides feedback on shooting and dribbling skills. The data collected can be sent to a smartphone app that helps players and coaches make adjustments to improve performance [14,15].
- American Football: Wilson X Connected Football: This smart football is equipped with sensors that track metrics such as speed, distance, spiral efficiency, and the catch/drop ratio. It is particularly useful for quarterbacks and coaches looking to fine-tune passing techniques [16].
- Golf: GEN i1 Smart Golf Ball: This ball comes with a built-in sensor that measures initial direction, speed, impact force, and rotation. Such data are crucial for golfers to understand and improve their stroke execution [17].
- Cricket: SmartBall by Kookaburra: This cricket ball has an embedded microchip that measures speed, spin, and power delivered by bowlers, providing valuable data for both players and coaches [18].
- Rugby: Adidas miCoach Smart Ball for Rugby: Similar to its soccer counterpart, this smart rugby ball is designed to provide kick data, including speed, spin, and the point of impact. This information is essential for improving the skills of place-kickers.
- Baseball: pitchLogic is a smart baseball designed to measure velocity, spin rate, and other pitching metrics directly from within the ball, providing real-time data that can be used to enhance pitching performance [19].

By leveraging sensor technology to provide real-time feedback and performance analysis, players can refine their techniques and enhance their overall proficiency. This integration of sensors into training not only empowers players to optimize their skills but also represents a significant advancement in the intersection of sports and technology.

### 2.2. Technology in Tennis

Correspondingly, an implementation of sensor fusion in tennis could be useful. There have been several instances of integrating technology and sensors into tennis, where most of the sensor integration is carried out on the racquet. Products such as the Sony Smart Tennis Sensor [20,21] and the Babolat Play racket with a Zepp sensor [22] are used an IMU for stroke detection. Such products extract data including stroke detection, stroke classification, ball impact location in the sweet spot, ball speed, and ball spin. From the data collected, researchers have concluded that junior players tend to use the forehand stroke potentially by gaining an advantage from a faster ball speed rather than having better hit variability. Having such sensors allowed coaches to access information in real-time and give advice to improve tactical–technical action aspects while in competition, showing the usefulness of meaningful real-time data being relayed for strategy/technique correction. However, reviews of these products indicate that attaching such a device to the butt of the racket increases its weight and leads to player discomfort due to its protrusion from the handle. Moreover, the sensor sometimes detaches during play [23].

Several current methods track high-speed sports. Hawkeye Innovations offers visual tracking through its TRACK system, which is widely used for ball trajectory tracking in tournaments and video assistant referee (VAR) calls. Their INSIGHT products align with the goals of this project. However, using Hawkeye’s high-speed cameras is expensive. In 2013, the English Premier League spent GBP 250,000 to install 14 cameras [24,25]. Given these costs, Hawkeye is predominantly feasible for tournament use rather than practice. This leaves a market gap for more affordable options in practice settings despite the demonstrated value of meaningful data from technologies like the miCoach ball and tennis racquet sensors.

### 2.3. Current Methods for Pose Estimation and Limitations

Most of the current technologies (excluding Hawkeye Innovations) use an inertial measurement unit (IMU), also known as a micro-electro-mechanical system (MEMS) IMU. This device uses mechanical properties to detect acceleration, rotation, and vibrations. Typically, an IMU includes an accelerometer for measuring acceleration along its reference axis and a gyroscope for detecting angular rates along the same axis. An IMU that combines these two sensors is referred to as a six-degree-of-freedom (6-DOF) IMU [26]. Both gyroscopes and accelerometers are prone to bias and noise, with gyroscopes experiencing drift due to bias instability and angular random walk high-frequency noise and accelerometers being sensitive to vibrations and non-gravitational accelerations.

To enhance accuracy, a magnetometer was added to create a nine-degree-of-freedom (9-DOF) IMU sensor. This sensor uses the Earth’s magnetic field in conjunction with a Kalman Filter for pose estimation [27]. However, magnetometers can be disrupted by strong nearby magnetic signals, which can overpower the Earth’s magnetic pull. This interference also complicates the calibration process of the magnetometer, as the Kalman filter relies on previous data for prediction, meaning that a sudden increase in external magnetic force can lead to incorrect predictions for several iterations. The use of Kalman filters in systems with multiple degrees of freedom is crucial for mitigating errors introduced by accelerometer and gyroscope drift. Therefore, sensor fusion for improving the accuracy and reliability of IMU readings is crucial.

### 2.4. Sensor Specifications

Based on the literature review, the target audience for our proposed electronic system includes junior and beginner tennis players who are learning the sport, as well as intermediate players aiming to increase their ball spin through progressive training. The product should feature a user interface that allows users to view data extracted from the sensor. Importantly, the entire product should ideally cost less than GBP 100. The displayed data should include RPM and the plane of rotation. The sensor should fit inside a tennis ball without altering the ball’s dimensions and the mass distribution within the ball should also be considered during assembly. Therefore, the specifications can be summarized as follows:

1. RPM data extracted from the tennis ball is accurate to ±5 RPM;
2. GUI is used to show data from the ball;
3. The ball fits within the weight and size dimensions to be an IPF-approved ball;
4. Mass distribution of the sensor system in the ball;
5. The sensor system costs less than GBP 100.

### 2.5. Tennis Ball Specifications

In this section of the methodology, we examine the specifications for tennis balls, as outlined by the International Tennis Federation (ITF) in the 2024 ITF Ball Approval Procedure. According to these guidelines, the diameter of a tennis ball should range from 6.54 cm (2.57 inches) to 9.00 cm (2.70 inches), and its mass from 25 g to 59.4 g, as detailed in Table 1 and Table 2. These parameters represent the allowable range for dimensions based on an extensive compilation of specifications.

**Figure 1:**
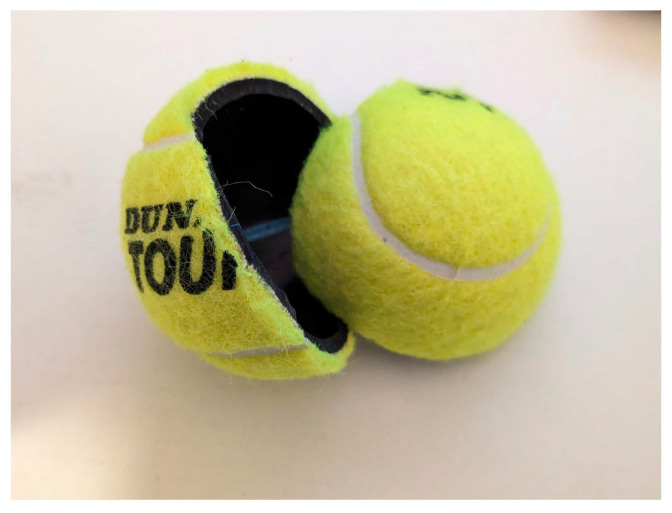
Cutting open a hollow-body pressurized tennis ball shows a layer of rubber inside the layer of felt. Reassembling the open ball will cause inconsistent surfaces, causing inconsistent rebounds.

The ITF recognizes two categories of tennis balls: pressurized and foam. Foam balls are specifically approved for tournaments involving players under ten years of age. A dissection of a standard pressurized tennis ball (Figure 1) reveals a felt layer and a rubber bladder, which is pressurized to ensure consistent rebound characteristics and deformation during play.

The Adidas miCoach smart ball features a design where sensors are suspended within the ball by rubber poles. This design, while effective for footballs, proves challenging for tennis balls due to their significantly smaller size, which is approximately 3.35 times smaller than a football. Implementing sensors and a power delivery system within these constraints requires careful design to maintain pressure and rebound consistency. Additionally, altering the tennis ball structure through cutting and resealing could cause deformations, leading to inconsistent rebound behavior.

**Figure 2:**
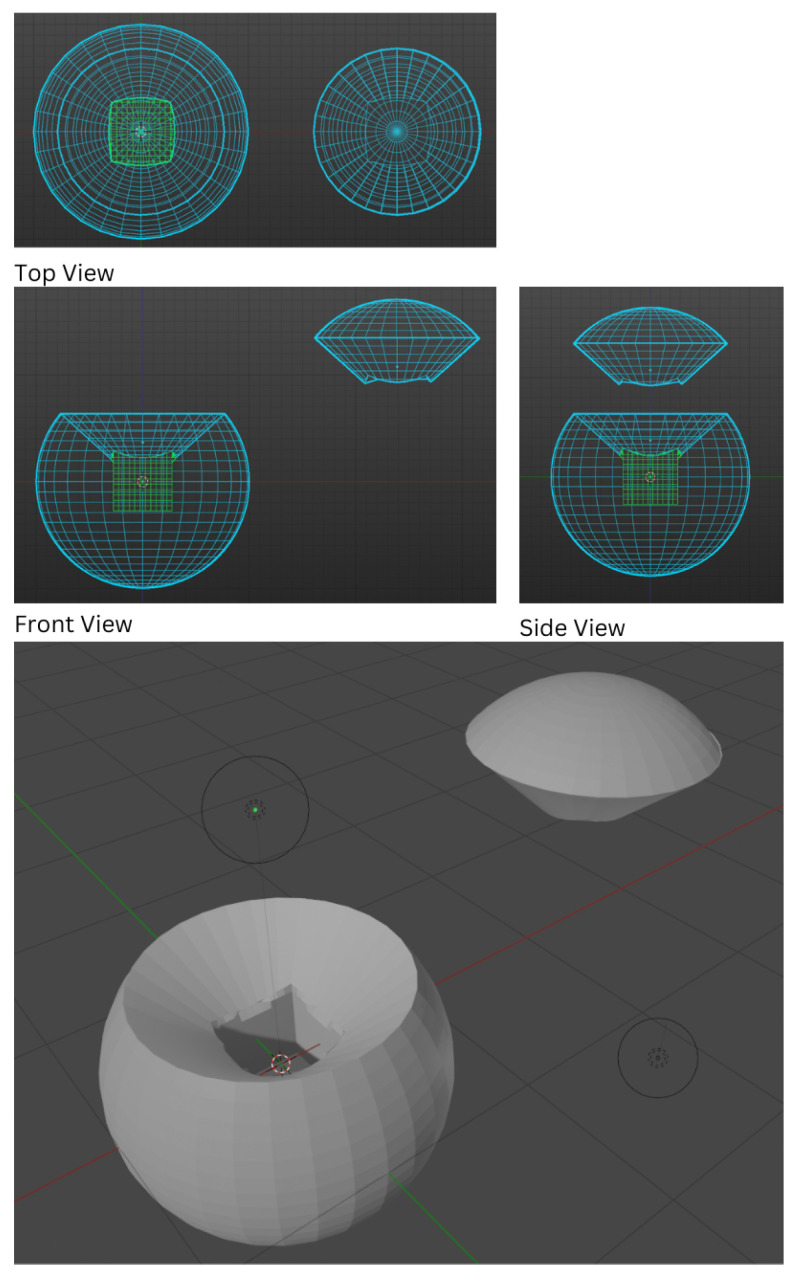
(**Top**) Orthographic view of a tennis ball with sensor pocket design highlighted in green; each side of the pocket is calculated using the equation derived from the constraint (2). (**Bottom**) Blender model of the tennis ball with a sensor pocket. Both were made with Blender [29].

Given these considerations, integrating sensors into a foam tennis ball is identified as a viable alternative, especially since the project targets youth players and beginners. Foam balls, certified by the ITF for use in under-10 competitions, conform to the necessary mass and size regulations. For this project, the Vermont foam tennis ball, certified for practice use, was selected in the 90 mm variant to allow ample room for sensor integration without significantly altering the ball’s integrity. The mass of the foam ball was measured as 43 g (as shown in Figure 2). Therefore, the density of the tennis ball, ρ , was calculated using the following formula:

(1) $$\rho_{\mathrm{ball}}=\frac{m_{\mathrm{ball}}}{\frac{4}{3}\cdot \pi \cdot {\left(\frac{d}{2}\right)}^{3}}$$

where *d* is the diameter of the tennis ball, which means the density was 0.113 mg mm^−3^. Since the pocket of foam for the sensor would need an equal distribution of mass, a cube is used in the middle offset. The following constraint is obtained (2):

(2) $$m_{\mathrm{sensor}}=\rho_{\mathrm{sensor}}\cdot l^{3}$$

where $m_{\mathrm{sensor}}$ is the mass of the sensor and power delivery system, and *l* is the maximum value between the height, width, and length of the system. After obtaining *l*, the foam tennis ball can be cut conically to reach the center of the ball, and the cubic section of side length, *l* can be removed from the middle. Figure 2 shows 3D models made with Blender showing the build configuration of the sensor pocket in the ball.

In designing the sensor integration, a preliminary model was developed featuring a sensor pocket, ensuring that the mass of the inserted sensor compensates for the removed foam volume. This approach is crucial for preserving the ball’s original mass and performance characteristics, which are essential for providing a consistent training experience for the target audience.

## 3. System Design

This section details the selection process and considerations for each hardware component: ball, power delivery system, and battery, as well as the essential components, system architecture, and the concept for the graphical user interface (GUI) of the tennis training ball. Drawing on insights from the literature review and the specifications outlined in Section 2.4, the augmented tennis training tool was designed primarily for beginner and youth players. Its goal is to enhance tennis strokes by providing quantifiable and meaningful feedback. Additionally, the tool was intended to be cost-effective and reusable for practice, offering a practical alternative to more expensive camera-based systems, which are typically better suited for tournament settings.

As described in Section 2.3, RPM data and the pose of the tennis ball are calculated by the IMU sensor. Currently, the system incorporates the BNO055 smart sensor fusion chip from Bosch, providing an integrated solution for pose estimation. The initial hardware setup included the Seeed XIAO nRF52840 Sense, paired with the Adafruit Triple-axis Magnetometer MMC5603 for sensor fusion. The XIAO nRF52840 Sense is equipped with an LSM6DS3TR-C 6-DOF IMU.

### 3.1. Block Diagram and Components

**Figure 3:**
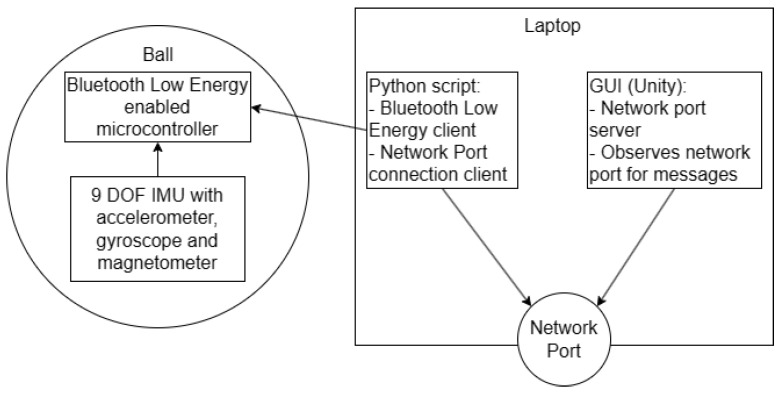
Block diagram of the high-level overview of components. (**Left**) Sensor component required in the tennis ball. (**Right**) Software components for data extraction and communication using BluetoothLE and a network port.

The system architecture is illustrated in Figure 3, depicting the minimal functional requirements for each component. The microcontroller, embedded within the ball, supports Bluetooth low energy (BLE) and interfaces with a 9-DOF MEMS IMU. A Python script functions as the BLE client due to the absence of a standard Bluetooth library for C# in Unity [30]. The data from the microcontroller were relayed to the Unity application via a network port on a Bluetooth-enabled laptop.

### 3.2. Interfacing between Components

**Figure 4:**
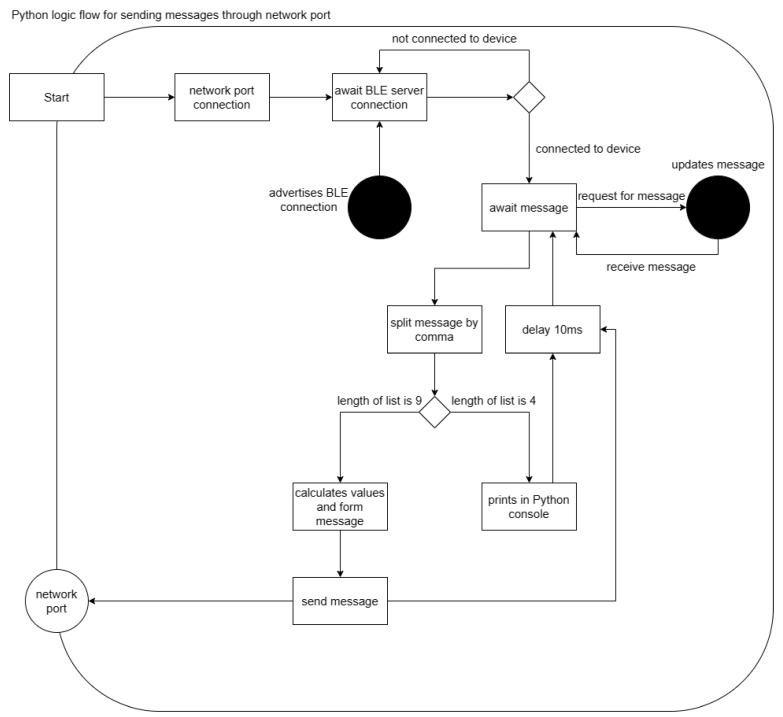
Python logic flow for sending messages to network port server in C# Unity, showing the difference in the message sent to Unity based on the length of the list obtained by splitting the comma delimited message from BluetoothLE.

To simplify the transition between initial and final designs, a “black box” interface method was used, focusing solely on the input and output to verify code correctness. This interface facilitates the data flow from the microcontroller to Python via Bluetooth and subsequently to C# through a network port, as shown in Figure 4. The communication protocol uses commas and semicolons to separate sensor state values. In Python, values were parsed using the split() and float() methods, with the array length indicating the state. In C#, the Split() method distinguishes between Vector3 objects and float values based on semicolon-separated indices. If the array length is 9, then a message will be formed and sent back to the network pot. On the other hand, if the length of the list is 4, it indicates a calibration state which is not relayed to the network port.

**Figure 5:**
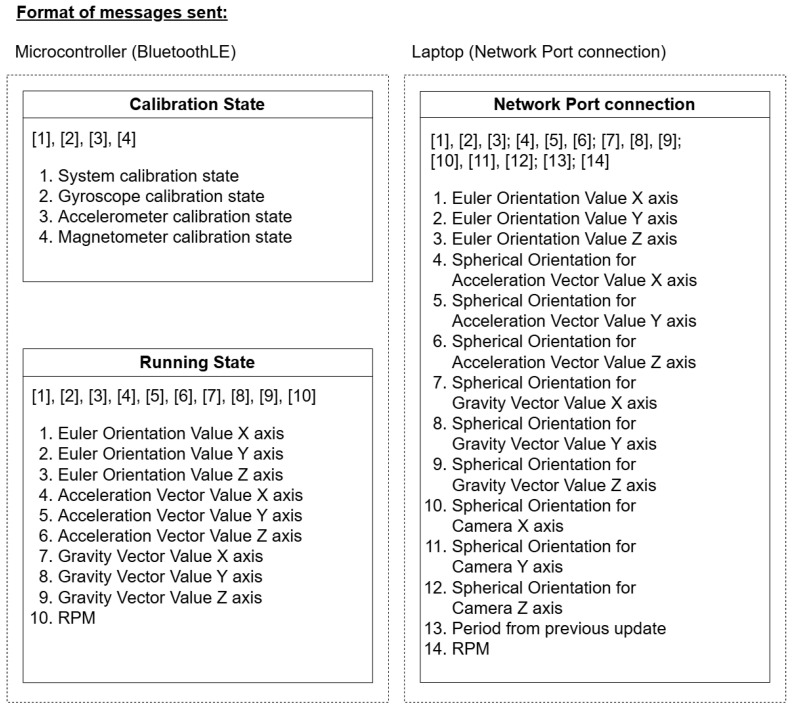
Format of messages between the sensor to python and python to the network port (C# Unity). (**Left**) From the sensor to Python [31] through Bluetooth [32] with 2 states of the microcontroller, showing comma-delimited messages with different lengths. (**Right**) From Python to C# Unity, showing semicolon-delimited and comma-delimited messages for quick processing in C#.

**Figure 6:**
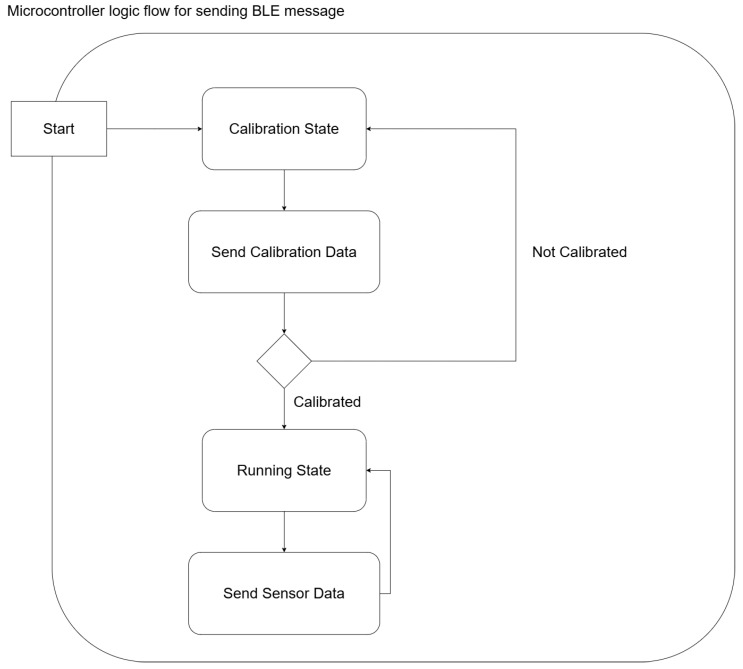
Microcontroller logic flow diagram for sending BLE messages to the client in Python, showing calibration and running state, where the format of data sent through BluetoothLE for each state is different.

Figure 5 demonstrates message formatting for efficient data transfer between components. The microcontroller operates in two modes: calibration and running. During calibration, it transmits detailed sensor state data for system-wide calibration; while in running mode, it sends refined sensor data, including orientation, acceleration, and RPM, all formatted to a precision of four decimal places and capped at 70 Bytes per message, as shown in Figure 6.

### 3.3. Power Delivery

A power delivery system is needed to power up the microcontroller and sensors while in the ball. The biggest constraint here is that the power delivery should fit inside the sensor pocket and weigh little so as not to need a larger hole to be cut as the sensor pocket. When evaluating possible batteries that could be used, some requirements regarding the battery became clear. The battery needed to be rechargeable, small, light, able to support at least 3 h of constant power supply, and ideally hot-swappable. A lithium polymer ion (LiPo) battery was chosen as the battery to be used. Compared to other batteries, the LiPo battery has more flexible sizing when compared to an AAA battery. The LiPo has a range of sizes that vary with capacity. Realistically, when a battery meets a certain capacity, the extra capacity can be considered a waste of space, which could constrain the size of the sensor system. The button cell typically uses a RTC module that might add mass and size to the overall system; in general, the button cell is also less hot-swappable since the LiPo battery is only connected using a JST connector.

### 3.4. Hardware

As previously mentioned, the sensors need to be lightweight, small, and cheap, and the microcontroller needs to be Bluetooth-enabled. Therefore, the chosen hardware was as follows:

1. Seeed Studio XIAO nRF52840 (Microcontroller): GBP 10.80;
2. SparkFun LiPo Charger/Booster, 5V/1A (Voltage regulator): GBP 13.80;
3. EEMB 3.7 V Li-ion 502030 Battery 250 mAh Lipo Battery (Power Supply): GBP 6.99
4. Adafruit BNO055 (inertial measurement unit): GBP 30.

The total cost for the sensor came to GBP 61.59, where the price for each of the components was a single item purchase. All items fit within the tennis ball and are considerably light, as listed in Figure 3. The Seeed XIAO nRF52840 uses the nRF52840, which has built-in Bluetooth connectivity while having a small form factor; it also has a 5 V IO power delivery pin and enough GPIO pins for communication between the IMU. Adafruit BNO055 uses Bosch’s fusion sensor technology that calculates the current orientation of the sensor to the world, gravity vectors in the XYZ axes for the sensor, and raw acceleration data. The LiPo charger Booster works with the LiPo battery to deliver 5 V power input to the Seeed XIAO nRF52840 and Adafruit BNO055. The LiPo charger Booster also acts as a way to charge the LiPo battery through a micro USB cable, and the battery can also be charged with the USB C input of the Seeed XIAO nRF52840. The charger booster is connected to the battery through the use of a JST connector, which allows LiPo batteries to be easily hot-swapped.

### 3.5. Software: GUI

This section covers the use of Unity and C# in conveying sensor information and orientation to the user, as well as setting up communication between the sensor and Python or C#.

**Figure 7:**
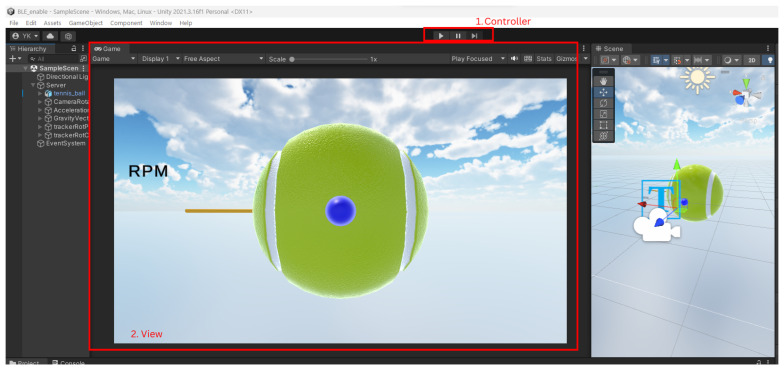
View and controller components of the MVC design pattern in Unity. View represents the frontend shown to the user. Controller represents how a user could interact with the GUI; for this GUI, the only input user available is to start the program.

The model–view–controller design pattern is a commonly found pattern when designing GUI [33]; it focuses on one of the best practices in design principles: separation of concern. In this case, the model is the sensor, Python, and simulated results in C#; the controller is back-end C# scripts in Unity; the view is the scene in Unity. Separation of concern, which can be seen in the model, is only used in the GUI to supply data. The controller receives user input to start the program, and the view only displays the program. All processing and calculation of data is carried out in the model and is not tied to processes in the controller and view. The controller and view is built into Unity. For the controller, input from the user is taken when the user starts the program. For the view, Unity starts a game window when an input is taken to start from the controller. Figure 7 shows the view and controller components as mentioned previously.

The GUI was designed to take into account how the user will associate the orientation with which a ball is spinning. In a typical match, the player receives the ball from where the acceleration of the ball is directed mostly toward the player instead of against the player. For example, a player will see a forehand stroke with a top spin as a stroke that spins from the top to the bottom instead of from the bottom to the top, which is how the player who stroked the ball would see it. However, keeping track of visualizing the rotation of the ball based on the negative unit vector direction of acceleration will not always show the right spin. For example, fixing an angle, δ , on the camera pointing at the negative unit vector direction of acceleration will result in seeing an opposite spin when the angle on the camera is rotated to δ + 180°. The gravity vector is used to determine the optimal δ . When thinking about the player looking at the ball, the eyes are oriented to see the gravity vector acting in the ball’s downward direction due to the player being affected by gravity acting downwards when standing upright. Assume the orientation of the ball is shown by rotating the ball based on the absolute orientation given by the Bosch sensor. It is important to acknowledge the fact that the absolute orientation of the ball does not need to be upright at initialization since the view showing the rotation of the ball only needs to convey the direction of spin of the ball over time, while the orientation of the camera is determined by the acceleration, gravity vector, and δ .

### 3.6. Communication

Bluetooth low energy (Bluetooth LE), also known as Bluetooth 4.0, was selected as the wireless communication medium between the sensor system and the laptop due to its energy efficiency. Compared to classic Bluetooth, Bluetooth LE consumes significantly less power, which helps conserve energy and reduces the frequency of battery recharges required for the sensor system. In the final product configuration, the sensor system functions as the Bluetooth LE server, while the laptop acts as the Bluetooth LE client, searching for and connecting to the sensor system.

### 3.7. Overall System

**Figure 8:**
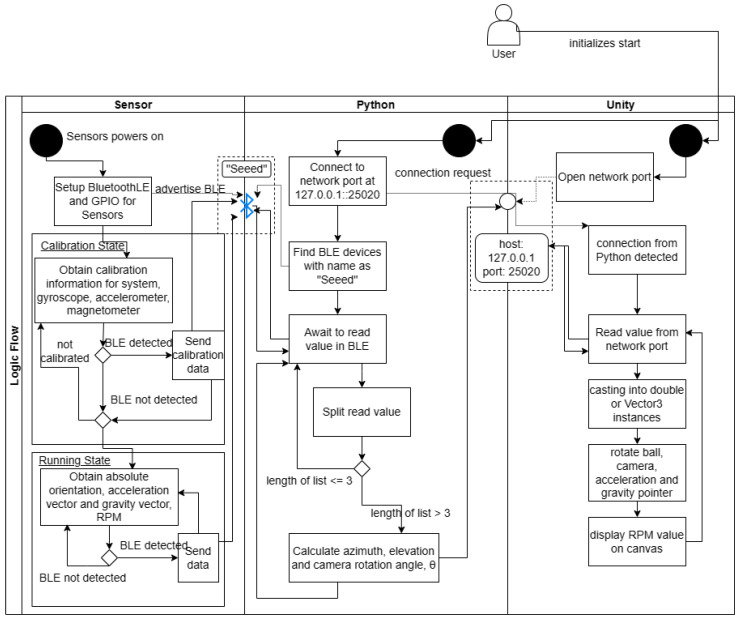
Full logic flow diagram of the final product, including the initialization of communication channels, and logic flow in messages sent. The black circle represents the start of each component. The user needs to initialize the start for the Unity and Python script subsequently for it to run, and the sensor system can be initialized anytime before the Python script, being a standalone unit.

Figure 8 shows the communication between components and the sequence in which communication needs to be established. The sensor works as a completely standalone unit; after every iteration, it checks if there is an identified client device. It sends data by updating the data that the client device can read. Unity opens an open listening server at the network port on the localhost (127.0.0.1) and waits for a connection to be made. The Python script starts a connection with the localhost server opened by Unity and finds BluetoothLE devices with a unique name before sending generic attribute (GATT) profiles to it to be identified. The sequence to start each component would be to start either the sensor or Unity and start the Python script last. This is because the Python script relies on the localhost server to be started and the BluetoothLE server with the unique name to be advertised.

## 4. Methodology and Implementation

This section of the article outlines the hardware and software development of the final product. Initially, we focused on improving how different sensors worked together, a process known as sensor fusion. Next, effort was devoted to the development of the graphical user interface (GUI). This interface is aimed at enabling users to visualize the rotation of the ball within a virtual environment provided by Unity.

### 4.1. Sensor Integration inside the Ball

**Figure 9:**
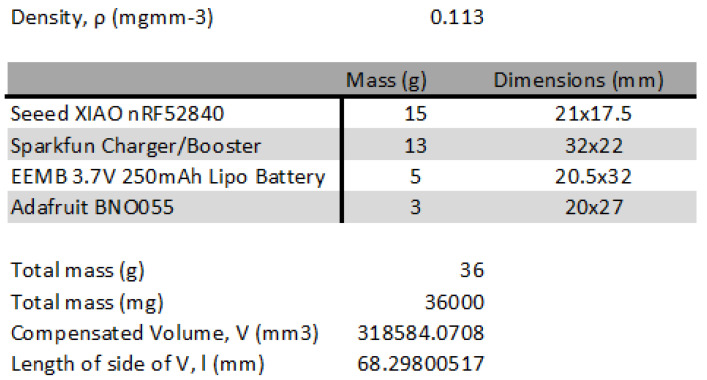
Calculation of sensor system pocket size, essential for calculating access weight when sensor pocket is cut out.

Figure 9 shows the mass and dimensions for each component as well as the calculation for the size of the pocket for mass compensation. The side length of the cube to remove from the tennis ball is 69 mm. By aligning the sensor system on the longer side, the dimension of the sensor system turns out to be 32 mm × 22 mm × 20 mm. The sensor system has a dimension that fits inside the pocket, but removing 69 mm is not feasible. By calculating the length of the furthest point of the pocket from the middle of the ball using the Pythagoras theorem, it can be seen that an isosceles triangle of side 34.5 mm provides a hypotenuse of 48.8 mm, which is more than the radius of the tennis ball.

**Figure 10:**
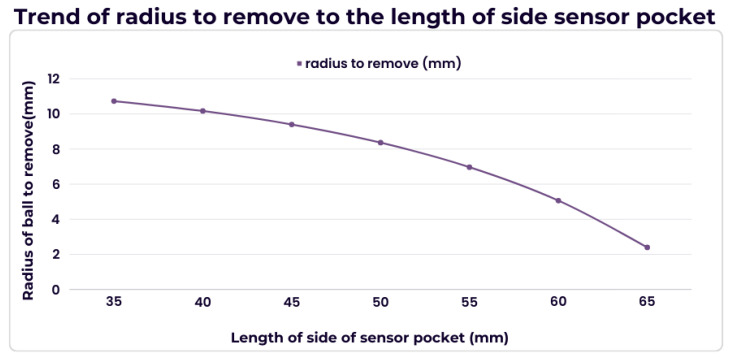
Trend of the radius of the tennis ball for the same rotation inertia as sensor pocket dimension increases. As the length of the side of the sensor pocket increases, the mass of the sensor system with the ball decreases since a larger portion of the foam is taken out.

By evaluating spin generation and the kinematics of the tennis ball. Spin is generated as a return stroke that applies a component of force that acts tangential to a point on the ball. In layman’s terms, rotational inertial (moment of inertia), *I*, describes how the ball reacts to the tangential component of the force. As rotational inertia increases, the tangential force needed to be applied for the object to be at a certain angular speed from stationary is higher. Angular speed dictates the current RPM, and rotational inertia dictates how much force needs to be exerted to reach a certain RPM. Rotational inertia is provided by $I=k\cdot m\cdot r^{2}$ , where *k* is the coefficient based on the shape of the rotating body, *m* is the mass of the rotating body, and *r* is the distance to the axis of rotation. In this case, *r* is 45 mm, and *m* is 43 g. Therefore, mass is inversely proportional to $r^{2}$ . By reducing *r*, it is possible to make the heavier ball have the same spin as a ball that meets the requirements but has a heavier feel than an ITF-certified ball. This trend is shown in Figure 10, extracted from data shown in Table 3.

The proposed method for integrating the sensor system into a tennis ball has several drawbacks, primarily due to the manual shaping of the sensor pocket. Carving the pocket by hand may lead to inconsistent results, as variations in the shaven radius can affect the ball’s rebound characteristics. Furthermore, using a cube-shaped pocket contributes to this inconsistency; the uneven thickness of the foam around the cube can result in varied rebound behaviors across different parts of the ball. Despite these challenges, the cube shape was selected because it is the simplest form to manually carve into foam.

To optimize the design, we integrated the charger booster, BNO055 sensor, and nRF52840 chip into a single board and constructed the ball around this consolidated sensor system. This approach minimized the mass and dimension of the sensor setup, potentially enhancing the consistency and performance of the ball.

**Figure 11:**
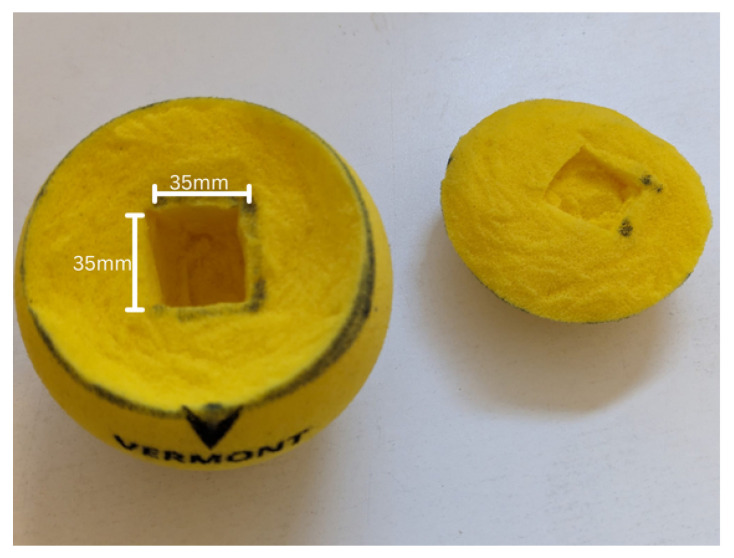
Sensor pocket cut out from the IPF-approved Vermont 90 mm foam tennis ball, showing pocket side length of 35 mm being cut out from the tennis ball.

For the current project, there was a compromise regarding the tennis ball’s weight since the sensor pocket measured 35 mm per side, resulting in an added mass of 31.3 g, as shown in Figure 11. This figure also illustrated the tennis ball with the sensor pocket carved out, highlighting the modifications made to accommodate the sensor system.

**Figure 12:**
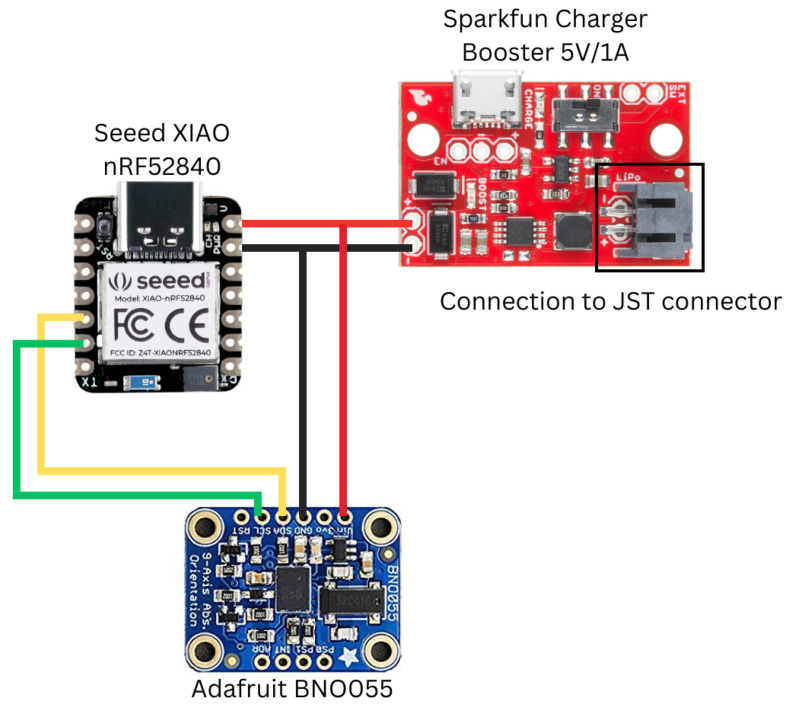
Overview of the connection between hardware components for the sensor system, including the connection of the Adafruit BNO055 to the SCL (green line, to pin P0.05 on Seeed) and SDA (yellow line, to pin P0.04 on Seeed) pins of the Seeed XIAO nRF52840, and connecting the 5 V power supply pin (red line) and ground (black line) of the Sparkfun Charger Booster to the 5 V input and ground pins of both the Seeed XIAO nRF52840 and Adafruit BNO055.

**Figure 13:**
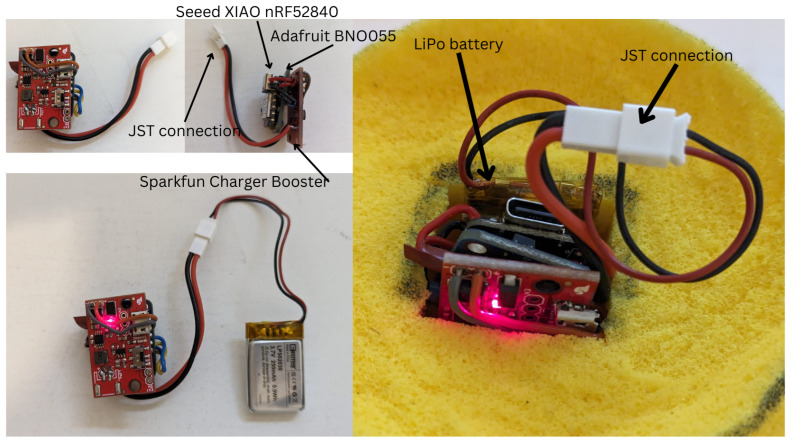
Implementation of a connection between hardware components for the sensor system.(**Top Left**) The connected sensor system, as shown in Figure 12, which is layered on top of another. The JST labeled connection is not connected to a LiPo battery. (**Bottom Left**) The connection of the sensor system to a battery, with a red LED showing that the power supply is working. (**Right**) The sensor system fitting inside the tennis ball, with the JST connector coming out of the pocket for quick and easy swapping of LiPo batteries.

The connection between components was made, as shown in Figure 12. Each board used in the sensor system was then stacked on top of one another to save space (Figure 13). The JST connector was soldered onto the highlighted region on the charger/booster. The LiPo battery was then connected to the JST connector. The JST connector dongles out of the sensor pocket in the tennis ball and allows for easy and quick swap between LiPo batteries, as shown in Figure 13.

### 4.2. Software: GUI

**Figure 14:**
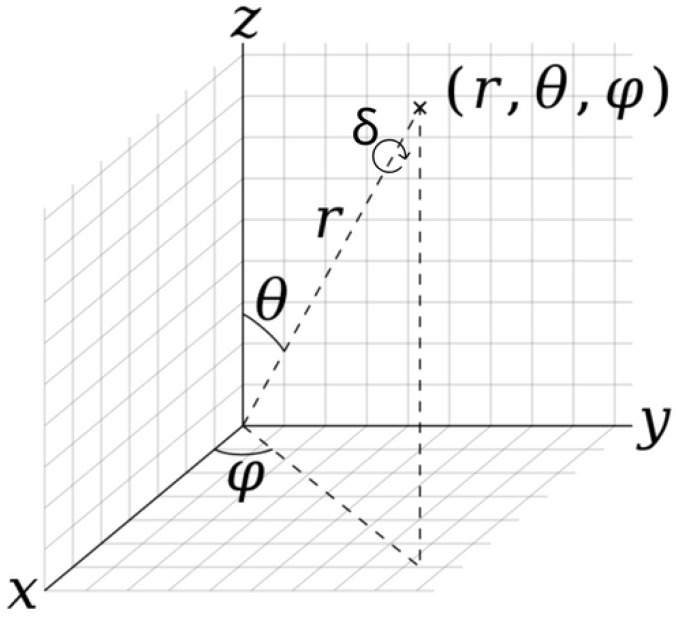
Spherical coordinates with an additional clockwise rotation angle δ that represents the rotation of the particle on the plane with a normal represented by vector from origin to particle at r, θ , φ , where r is the radial distance, θ is the polar angle, and φ is the azimuth angle.

The rotation of the tennis ball is of vital importance for the players in the training process. However, it should be noticed that the interpretation of how a body is rotating is solely based on the perspective of the viewer. To avoid any ambiguity, the camera angle needs to be constrained. A simple example can be given to explain this phenomenon. Set the camera constantly pointing towards the origin/ball, and assume the ball is spinning clockwise on the y-axis, as shown in Figure 14. Variables r, θ , and φ represent the position in spherical coordinates, and δ shows the orientation of the camera. A camera with constant δ = 0, at (r, 90, 0) will see a backspin. However, by changing the position to (r, 90, 180), the camera will see a topspin. Also, if the variable δ changes from 0 to 180, the direction of rotation seen by users would also be completely opposite. Section 3.5 hypothesizes that a user commonly regards the spin of the ball when the ball is returned and is accelerating towards the general direction of the player. In order to facilitate the player’s observation, set the camera point in the opposite direction of the acceleration vector acting on the ball and the camera orientation to be relatively downward. This could ensure that the camera views the ball from the right side up.

However, Unity does not use spherical convention. GameObjects rotate using Euler angles and translate (change of position) using a left-handed coordinate system. Therefore, the translation of the camera relative to the ball would be the unit vector of the acceleration vector. Instead of calculating Euler angles, the spherical coordinate convention could be recreated on the camera by offsetting the camera from the origin inside a parent GameObject and rotating the parent gameObject using Euler angles. By translating the MainCamera GameObject by [0, 0, 0.5], the polar angle θ could be given by a rotation on the x-axis, and the azimuth angle φ could be given by a rotation on the y-axis. It also means that the vector representation of θ=90 and φ=0 corresponds to unit vector [0, 0, 1] (z-axis, assuming the positive direction); θ=0 corresponds to unit vector [0, 1, 0] (y-axis); the vector representation of θ=90 and φ=90 corresponds to unit vector [1, 0, 0] (x-axis).

As mentioned in Section 3.5, the acceleration vector and gravity vector are used to calculate the orientation of the camera. The sensor data provided by Adafruit for acceleration and gravity are three-dimensional vectors. The values for θ and φ are calculated from these values in Python using basic trigonometry. The equations are as follows:

(3) $$\vec{g}=\left[g_{x},g_{y},g_{z}\right]$$

(4) $$h_{g}=\sqrt{g_{x}^{2}+g_{z}^{2}}$$

(5) $$\varphi =\arctan(\frac{g_{x}}{g_{z}}),$$

(6) $$\theta =\left\{\begin{array}{cc} -\tan^{-1}\left(\frac{|g_{y}|}{|h_{g}|}\right), & \mathrm{if}\;g_{y}\geq 1\\ \tan^{-1}\left(\frac{|g_{y}|}{|h_{g}|}\right), & \mathrm{otherwise} \end{array}\right.$$

φ:[−180,180]

θ:[90,−90]

**Figure 15:**
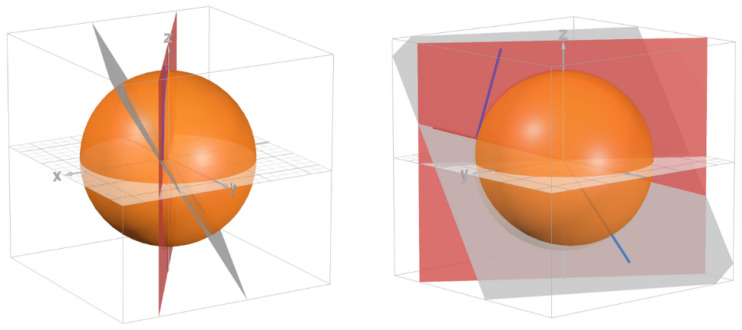
(**Left**) Plane of $\vec{a}$ and the z-axis shown in red with $\vec{c}$ being inside the plane and plane of $\vec{a}$ and $\vec{g}$ shown in grey. (**Right**) Side view showing both planes intersecting on the axis of rotation.

From the above expressions, $\vec{g_{y}}$ represents the gravity vector. The camera was fixed on the ball with one unit vector of the acceleration vector away from the ball. Figure 15 shows the validity of using two planes to find δ : plane A contains $\vec{a}$ and $\vec{g}$ while plane B contains $\vec{a}$ and z-axis. Here, $\vec{a}$ and $\vec{g}$ are assigned values as an example to better explain the process:

(7) $$\vec{a}=\left[\begin{array}{c} 2\\ 3\\ 1 \end{array}\right]$$

(8) $$\vec{g}=\left[\begin{array}{c} -3\\ -1\\ -4 \end{array}\right]$$

Here, the acceleration vector, $\vec{a}$ , and the gravity vector, $\vec{g}$ , are represented as a red and a blue line, respectively. The camera orientation, $\vec{c}$ , is represented as a purple line. $\vec{c}$ rotates by δ on the red plane to align on the same plane with $\vec{a}$ and $\vec{g}$ , where $\vec{c}$ is always tangential to $\vec{a}$ . Since $\vec{a}$ is the axis of rotation, the plane with $\vec{a}$ and $\vec{c}$ , and the plane with $\vec{a}$ and $\vec{g}$ can be compared to obtain δ . However, this is not feasible as $\vec{c}$ is not a vector obtained from the sensor system and is instead the product of rotating the parent GameObject of the camera by θ and φ .

Equations of planes are obtained using the cross-product of two vectors to determine the normal line from the plane. The cosine value for both normal lines can be used to calculate the reference angle subtended between the planes.

(9) $$\vec{z}=\left[\begin{array}{c} 0\\ 0\\ 1 \end{array}\right]$$

(10) $$\vec{N_{\mathrm{AZ}}}=\left|\begin{array}{ccc} i & j & k\\ 0 & 0 & 1\\ 2 & 3 & 1 \end{array}\right|=\left[\begin{array}{c} 3\\ -2\\ 0 \end{array}\right]$$

(11) $$\vec{N_{\mathrm{AG}}}=\left|\begin{array}{ccc} i & j & k\\ -3 & -1 & -4\\ 2 & 3 & 1 \end{array}\right|=\left[\begin{array}{c} -11\\ 5\\ 7 \end{array}\right]$$

(12) $$\delta_{\mathrm{ref}}=\cos^{-1}\left(\frac{\vec{N_{\mathrm{AZ}}}\cdot \vec{N_{\mathrm{AG}}}}{|\vec{N_{\mathrm{AZ}}}\left|\cdot \right|\vec{N_{\mathrm{AG}}}|}\right)$$

From expression (9), $\vec{z}$ represents the unit vector along the z-axis. Moreover, $\vec{N_{\mathrm{AZ}}}$ represents the normal line of the plane that encapsulates $\vec{a}$ and $\vec{z}$ , and $\vec{N_{\mathrm{AG}}}$ represents the normal line of the plane that encapsulates $\vec{a}$ and $\vec{g}$ .

Furthermore, $\delta_{\mathrm{ref}}$ provides the reference angle for calculating δ as the direction of the gravity vector is lost when using the plane to calculate $\delta_{\mathrm{ref}}$ . Two-dimensional trigonometry uses reference angles as the smallest acute angle from the $0^{{}^{\circ}}/180^{{}^{\circ}}$ axis. To resolve trigonometric calculations based on the quadrants of a circle, a similar process should be carried out in 3D (sphere). However, there are few studies regarding resolving reference angles in spheres. By approaching this problem as an extension of 2D trigonometry, the sphere is evaluated based on quadrants and split up into eight quadrants to determine patterns between the reference angle and quadrants. This step is carried out using Python with the library itertools to obtain all possible combinations of coordinates for values of −1, 0, and 1. With reference to $\vec{a}$ of [0, 0, 1], the list is then processed to calculate θ and φ , where angles to all quadrants and edge cases are obtained. $\delta_{\mathrm{ref}}$ is then calculated and compared visually in Unity.

**Figure 16:**
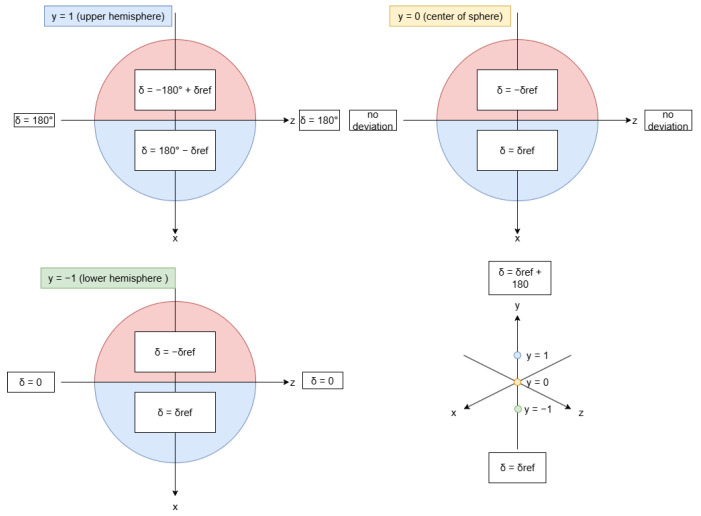
Illustration of regions of a sphere and the modifier to be applied to $\delta_{ref}$ to obtain δ . (**Top Left**) Modifier for each quadrant of the upper hemisphere. (**Top Right**) Modifier for each quadrant of the center of the sphere (equator). (**Bottom Left**) Modifier for each quadrant of the lower hemisphere. (**Bottom Right**) Modifier for the top and bottom of the sphere, also shows color-coded spots of where each of the diagrams lies on the 3D cartesian.

The results are shown in Table 4 and illustrated in Figure 16. Therefore, the result of δ is compiled into the following cases:

(13) $$\delta =\left\{\begin{array}{cc} +\delta_{\mathrm{ref}}, & \mathrm{if}\;90<\theta <180,\;0<\varphi <180\\ -\delta_{\mathrm{ref}}, & \mathrm{if}\;90<\theta <180,\;-180<\varphi <0\\ 180-\delta_{\mathrm{ref}}, & \mathrm{if}\;0<\theta <90,\;0<\varphi <180\\ -180+\delta_{\mathrm{ref}}, & \mathrm{if}\;0<\theta <90,\;-180<\varphi <0\\ 0, & \mathrm{if}\;90<\theta <180,\;\varphi =0/180\\ 180, & \mathrm{if}\;0<\theta <90,\;\varphi =0/180\\ 0, & \mathrm{if}\;\theta =180,\;-180<\varphi <180\\ 180, & \mathrm{if}\;\theta =0,\;-180<\varphi <180 \end{array}\right..$$

According to the above expressions, δ is calculated in Python and sent to C# Unity with the X and Y rotation floats for the acceleration vector as the rotation Vector3 for CameraRotation. Since δ is calculated by comparing the difference in θ and φ from the acceleration vector, this value needs to be calculated before using Equation (13) as the range of values for the difference in θ and φ ranges from [0, 180] and [−180, 180], respectively.

### 4.3. Communication

This section details the implementation of the Bluetooth low energy (BluetoothLE) server on a microcontroller using embedded C and the setup of the BluetoothLE client in Python. For the microcontroller, the ArduinoBLE.h library facilitated the creation of a BLEService and a BLEStringCharacteristic using specific UUIDs for service and characteristic:

- Service UUID: 19b10000-e8f2-537e-4f6c-d104768a1214;
- Characteristic UUID: 19b10001-e8f2-537e-4f6c-d104768a1214.

These UUIDs helped define the type of service and data characteristic, with the characteristic supporting a maximum message size of 70 bytes, although up to 150 bytes were set as acceptable to accommodate larger data values. The microcontroller was configured to allow client devices to read data, which involved initializing BluetoothLE, setting the device name to "Seeed", adding the characteristic to the service, and advertising the service.

On the Python side, the bleak library was used to interact with BluetoothLE. It scanned for and connected to the microcontroller by matching the device name "Seeed". Using the same characteristic UUID as the microcontroller, the Python script could read the transmitted data, which were parsed as specified in another section of the document.

**Figure 17:**
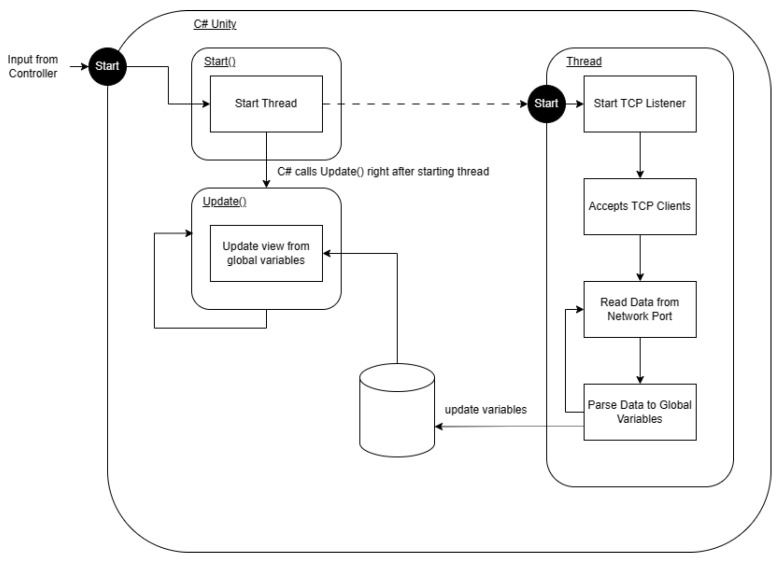
Thread used in setting up TCP connection through the network port with C# Unity as the server. The thread waits for a connection from the untimely connection from Python without interfering with the Update() function.

Next, we set up communication through a network port between Python and C# in Unity, facilitated by a TCP connection on the same machine. A listening server was implemented in C# Unity using the System.Net.Sockets library, where a TCP listener was initiated with TcpListener(IPAddress.Any, connectionPort). This setup allows acceptance from both IPv4 and IPv6 addresses and sets the network port number as specified in connectionPort, an integer provided via the Unity GUI. The server was activated with server.Start(), and upon receiving a connection request from Python, it accepted the client using server.AcceptTcpClient(). To ensure reliable connections, the server setup and client connection processes were executed within a separate thread, as depicted in Figure 17. This threading allowed Unity’s Update() function to run uninterrupted while the server waits for connection requests and data from Python.

In Python, the socket library was used to send a TCP connection request. A socket instance was created with socket.socket(socket.AF_INET, socket.SOCK_STREAM), indicating use of IPv4 addresses and a TCP type connection. Connection to the C# Unity server was established using sock.connect((host, port)), with host set to the localhost IP address (127.0.0.1) and port matching the Unity-specified port (25020).

### 4.4. Tennis Ball Characteristics

This section covers the implementation of the graphical user interface (GUI) in Unity, as discussed in Section 3.5, which discusses how users could identify the type of spin on a ball. The system used the acceleration vector, gravity vector, and the ball’s rotation relative to the world axis to determine the optimal camera orientation for viewing and classifying spin.

**Figure 18:**
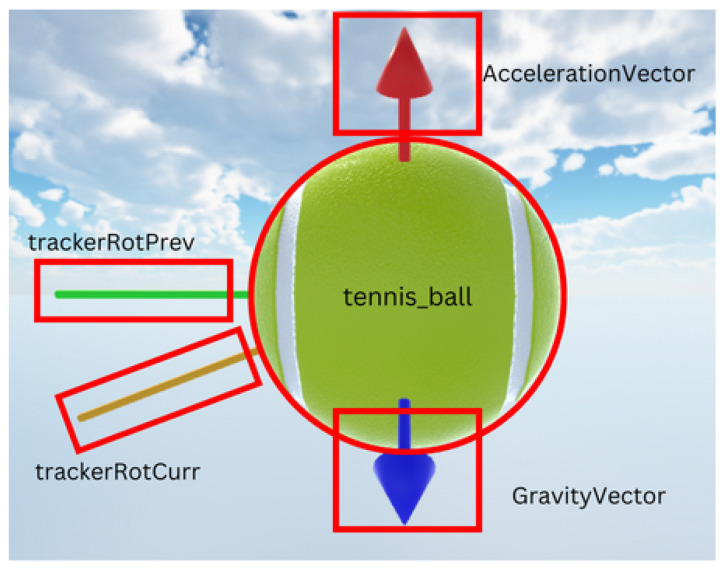
GameObject in a Unity scene with the use of trackerRotPrev and trackerRotCurr during implementation.

In Unity, GUI elements were managed by a script named BLE.cs, which was attached to the ‘Server’ parent GameObject. The orientation of the tennis_ball GameObject aligned with the absolute orientation data from the BNO055 sensor. Additionally, the AccelerationVector and GravityVector were visual pointers that indicated the directions of acceleration and gravity acting on the sensor. A cameraRotation GameObject, not shown in Figure 18, controlled the user’s viewpoint within the scene.

The camera was oriented towards the ball at the origin, translating spherical coordinates (r,θ,φ) into Unity’s Euler angles. This was achieved by adjusting a parent GameObject’s angles in Unity, using acceleration and gravity data from the Adafruit sensor to calculate θ and φ via Python’s trigonometric functions, ensuring accurate visualization of the ball’s spin.

The tennis ball’s RPM was calculated based on the gyroscope’s angular velocity using the following equations:

(14) $$\omega_{\mathrm{resultant}}=\sqrt{{\mathrm{gyro}}_{X}^{2}+{\mathrm{gyro}}_{Y}^{2}+{\mathrm{gyro}}_{Z}^{2}}$$

(15) $$\mathrm{RPM}=\frac{\omega_{\mathrm{resultant}}\cdot 60}{2\cdot \pi }$$

From the above epxressions, ${\mathrm{gyro}}_{X}$ , ${\mathrm{gyro}}_{Y}$ , and ${\mathrm{gyro}}_{Z}$ represent the angular velocity along the x, y, and z axes. $\omega_{\mathrm{resultant}}$ is the combined angular velocity, calculated as the square root of the sum of the squared angular velocities.

The sensor system computed this equation, storing a buffer of previous RPM values in Python. The average of the last five values was used to smooth the data before sending the RPM through the network port to be displayed on the canvas GameObject. To address camera movements caused by jittery acceleration data at low speeds, acceleration values below 1 in each axis were filtered to zero, which stabilized the calculated θ and φ angles.

## 5. Results

As previously mentioned, the cost breakdown of each component can be found in Section 3.4. The sensor system costs GBP 61.59, and the Vermont tennis ball costs GBP 9.99, making the total cost of the final product GBP 71.58, which meets specification 5. Mass distribution was considered during the design phase (Figure 2), fulfilling specification 4. The graphical user interface (GUI), which displays the ball’s rotation and RPM, is shown in Figure 7, satisfying specification 2. However, specification 3 was not met, as the ball exceeds the weight range for a U10 tournament foam ball.

The tennis ball’s angular velocity was measured using gyroscopic sensors to calculate the rotations per minute (RPM). The Euler angles (X, Y, and Z axes) showed deviations due to misalignment of the sensor system. By analyzing these data, the rotation periods were highlighted with color coding to distinguish rotation states.

Table 5 demonstrates that the system accurately measured the ball’s RPM, which reached a maximum of 14 RPM, within the expected range when accounting for buffer smoothing. Orange-highlighted rows indicate no rotation, while yellow and green rows represent active rotation states.

From the data, the rotation period (T) was 1.68 s. The theoretical RPM, based on the calculation formula, was 17.86, while the maximum observed RPM from the sensor was 14, showing the system’s accuracy within a reasonable margin. Although there was a slight lag in RPM detection due to averaging, the system met the RPM specification 1 and accurately measured the tennis ball’s rotations.

To improve the GUI experience and reduce choppy ball rotation, changing the ball material and using Quaternion.Slerp in Unity is recommended. A video demonstration of the GUI is available on a YouTube playlist, showcasing the ball’s rotation and general movement.

**Figure 19:**
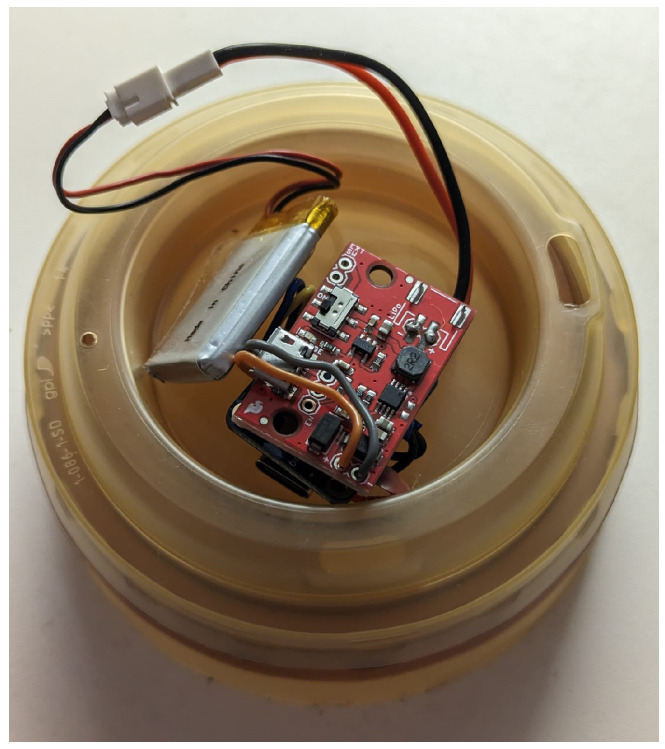
Sensor system on a rotating plane. The plane is rotated by approximately 180° by aligning the hole to the opposite of its original position.

To validate specification 1, the sensor system was removed from the tennis ball and placed on a rotating plane to evaluate the RPM output. A rotating plane was used because it is easier to trace the angle rotated than trying to rotate the sensor on a single axis. Rotating the sensor on a flat surface revealed angle deviations across several axes due to surface irregularities and imperfect sensor alignment. Using a rotating plane ensured the measured rotation represented the resultant angle deviation. Figure 19 shows the sensor system on a rotating plane, where the angle deviation was tracked by the position of a hole on the plane. The plane was then rotated 180°, aligning the hole directly opposite its starting position. The data were tracked and processed using Python.

The Euler angles for axes X, Y, and Z showed deviations due to the sensor system’s misalignment on a flat surface, causing an angle deviation of approximately 180°. Validation involved inspecting the time taken to rotate this angle, using the lack of deviation between Euler angles to denote the start and end of rotation. Table 5 shows a snippet of responses from the sensor system during detected rotations. Rows highlighted in orange indicate no rotation, while yellow and green highlight active rotation states. Yellow-highlighted rows indicate RPM values averaged with 0 values before rotation began. The green-highlighted rows show the maximum RPM of 14 before decreasing to 2 RPM as the rotation ends.

The rotation period (T) was 1.68 s. The theoretical RPM was 17.86, and the maximum observed RPM from the sensor was 14, which is within the expected range mentioned in specification 1.

Analyzing the rotation axis most affected by plane rotation, axis X had a rotation range of 4.4° to 174°. When evaluating the RPM for each timestamp, the yellow-highlighted region exhibited high angle deviation due to the buffer’s 0 values. This meant RPM would lag by approximately 0.5 s, but it did not significantly affect RPM prediction. The product met specification 1, as the tennis ball’s RPM remained consistent for longer than the validation time (1.68 s).

A video recording of the GUI is available on the following YouTube playlist: https://youtube.com/playlist?list=PL1HE9_VZc6AK6yN-dXTHPf7ha2_9l_aeH&feature=shared (accessed on 21 July 2024). It showcases the rotation of the ball in the GUI. Although the ball’s rotation appears choppy, its general direction can be observed. Changing the ball’s material in Unity and using Quaternion.Slerp for smoother animation may improve the results.

**Figure 20:**
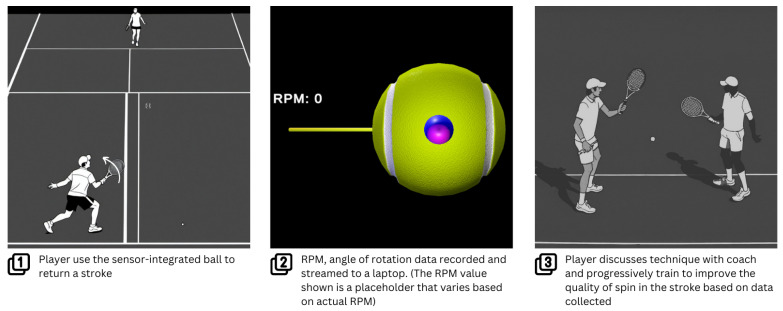
An example use case of the sensor-integrated tennis ball in for real-time feedback and performance analysis. Sub-figure showing steps 1 and 3 are generated using OpenArt AI. Step 1 shows a player performing a forehand stroke in tennis. Step 2 shows a user interface that provides real-time feedback on RPM, which can be used to represent a quantitative value that the player is trying to maximize in the forehand stroke. Step 3 shows the coach using the feedback provided to keep track of how the player’s stroke is improving while improving the technique of the player.

As for the use case of the ball, a player could leverage sensor technology to provide real-time feedback and performance analysis. Players can also refine their techniques and enhance their overall proficiency. This integration of sensors into training not only empowers players to optimize their skills but also represents a significant advancement in the intersection of sports and technology. A use case can be seen in Figure 20, where the player uses the RPM value extracted from a sensor-integrated tennis ball to be used in practice. This method of practice provides further insight for coaches to track improvement while a player develops their technique. Without this sensor technology, players and coaches need to evaluate technique based on feel, which is hard for an inexperienced player.

## 6. Discussion

Existing tennis sensor technology is mainly achieved through Hawkeye or integrated into the racket. Unlike previous methods, this study demonstrates an innovative approach by integrating smart sensors into tennis balls. It not only provides tennis players with a more convenient training tool but also offers new avenues for further development in smart tennis and sports analytics technology. By embedding sensors into tennis balls, key motion indicators such as ball rotation speed and acceleration direction can be monitored in real-time, providing players with instant feedback and personalized guidance to improve their tennis skills.

Despite showcasing the potential of sensor fusion technology in tennis training, there are some drawbacks to this study. For example, the ball’s mass is 31.3 g above the U-10 foam ball limit and it uses a foam ball instead of a pressurized ball. The foam ball is easier to reassemble than a pressurized ball, but the foam ball’s rebound is compromised by the non-spherical pocket inside, leading to inconsistent foam coverage. Also, the stability and reliability of the technology need further validation, especially in real-game environments.

Future research may optimize sensor integration solutions to address the issue of uneven material and deformation in tennis balls. Moreover, the sensor currently relies on batteries. To ensure further advancements and lessen reliance on batteries, which would also help reduce size, we will explore the possibility of a self-driven or autonomously driven system. For this, we are considering energy harvesting mechanisms that rely on the use of, for example, piezoelectric materials. These materials generate electricity through mechanical stress when the ball is hit [34]. Additionally, integrating virtual reality and augmented reality technologies can create more immersive training environments to further improve training effectiveness. Ultimately, sensor fusion technology will become one of the standard training tools for tennis in the future, with ongoing technological breakthroughs and cost reductions.

## 7. Conclusions

This article demonstrates the feasibility and implementation of integrating sensors into tennis balls for training purposes. The final product adheres to the ITF-approved size dimensions of 90 mm for U-10 Junior tournaments, although it exceeds the mass requirement by 31.3 g for a U-10 foam ball. The Bosch BNO055 sensor and its integrated sensor fusion software performed well, enabling the graphical user interface (GUI) to display data and calculate RPM. The entire sensor system costs significantly less than current methods, such as Hawkeye Innovations, at GBP 61.59. Additionally, data obtained from the Bosch BNO055 chip could be used to derive further parameters of the stroke, such as the force acting on the ball due to the Magnus effect using the RPM value. This technology also opens up possibilities for various applications, such as a VR game using an actual tennis racquet with the ball pivoted on a fixed arm. Real-life stroke data could then be realistically represented in the virtual environment.

Our prototype product resulted in several successful software implementations beyond the sensor system itself. The design provides an effective way to visualize ball rotation. Unlike current methods, the GUI not only shows the RPM but also displays the ball’s orientation. Additionally, we established wireless communication between the ball and the GUI. Although Unity C# traditionally lacks a direct method for communicating with Bluetooth sensors (with most data exchange occurring through the COM port), our successful implementation of this communication demonstrates a viable pathway for applications requiring wireless interaction between hardware and Unity using BluetoothLE.

However, there are some limitations, as highlighted in the article. The ball’s weight is 31.3 g above the limit for U-10 foam balls, and it uses a foam ball instead of a pressurized one. Although easier to assemble, the foam ball’s rebound quality is compromised by an internal non-spherical pocket, resulting in inconsistent foam coverage.

Nevertheless, this article demonstrates the feasibility of integrating sensors into tennis balls for real-time data collection and display. It presents the design and calculations required for a novel GUI that aligns the ball’s spin with the user’s perspective. The article also outlines a method for connecting an Arduino sensor to Unity C# via BluetoothLE and network ports, which could serve as a foundation for integrating wireless custom controllers, potentially offering a unique gaming experience.

## Figures and Tables

**Table 1:** Tournament tennis ball requirements from the International Tennis Federation for the year 2024, [28].

	TYPE 1 (FAST)	TYPE 2 (MEDIUM)	TYPE 3 (SLOW)	HIGH ALTITUDE
**MASS (WEIGHT)**	56.0–59.4 g	56.0–59.4 g	56.0–59.4 g	56.0–59.4 g
**SIZE**	6.54–6.86 cm	6.54–6.86 cm	7.00–7.30 cm	6.54–6.86 cm
**REBOUND**	138–151 cm	135–147 cm	135–147 cm	122–135 cm

**Table 2:** Tournament tennis ball requirements (U-10) from the International Tennis Federation for the year 2024, [28].

	STAGE 3 (RED) FOAM	STAGE 3 (RED) STANDARD	STAGE 2 (ORANGE) STANDARD	STAGE 1 (GREEN) STANDARD
**MASS (WEIGHT)**	25.0–43.0 g	36.0–49.0 g	36.0–46.9 g	47.0–51.5 g
**SIZE**	8.00–9.00 cm	7.00–8.00 cm	6.00–6.86 cm	6.30–6.86 cm
**REBOUND**	85–105 cm	90–105 cm	105–120cm	120–135 cm

**Table 3:** Data for calculation of the radius of the tennis ball for the same rotation inertia.

Length of Side Pocket (mm)	Total Mass of Ball & Sensor (mg)	Corresponding Radius r (mm)	Radius to Remove (mm)
**35**	74,155.1	34.3	10.7
**40**	71,768	34.8	10.168
**45**	68,702.9	35.6	9.4
**50**	64,875	36.6	8.4
**55**	60,199.6	38.0	6.968
**60**	54,592	39.9	5.1
**65**	47,967.4	42.6	2.4

**Table 4:** Result of the modifier applied to $\delta_{\mathrm{ref}}$ to align $\vec{a}$ and $\vec{g}$ on the same plane, with $\vec{g}$ pointing towards the bottom half hemisphere.

bX	bY	bZ	θ	φ	$\delta_{\mathrm{ref}}$	Modifier	δ
−1	−1	−1	35	−135	45	−deg	−45
−1	−1	0	45	−90	45	−deg	−45
−1	−1	1	35	−45	45	−deg	−45
−1	0	−1	0	−135	90	−deg	−90
−1	0	0	0	−90	90	−deg	−90
−1	0	1	0	−45	90	−deg	−90
−1	1	−1	−35	−135	45	−180 + deg	−135
−1	1	0	−45	−90	45	−180 + deg	−135
−1	1	1	−35	−45	45	−180 + deg	−135
0	−1	−1	45	180	0	+deg	0
0	−1	0	90	0	0	+deg	0
0	−1	1	45	0	0	+deg	0
0	0	−1	0	180	0	+deg	0
0	0	0	0	0	0	+deg	0
0	0	1	0	0	0	+deg	0
0	1	−1	−45	180	0	+deg + 180	180
0	1	0	−90	0	0	+deg + 180	180
0	1	1	−45	0	0	+deg + 180	180
1	−1	−1	35	135	45	+deg	45
1	−1	0	45	90	45	+deg	45
1	−1	1	35	45	45	+deg	45
1	0	−1	0	135	90	+deg	90
1	0	0	0	90	90	+deg	90
1	0	1	0	45	90	+deg	90
1	1	−1	−35	135	45	180 − deg	135
1	1	0	−45	90	45	180 − deg	135
1	1	1	−35	45	45	180 − deg	135

**Table 5:** Euler angle deviation represented by the sensor system, and RPM recorded from the sensor system.

TimeID	Cummulative Time (s)	eulerX	eulerY	eulerZ	RPM
1704721509.8699	0	4.8125	−7.9375	−13.25	0
1704721509.99058	0.1207	4.4375	−7.75	−13.0625	0
1704721510.11058	0.2407	10.9375	−7.9375	−12.5	1.4
1704721510.23092	0.3610	27.625	−8.125	−11.9375	3.4
1704721510.3503	0.4804	48.1875	−8.125	−11.375	6.6
1704721510.47071	0.6008	66.875	−8.0625	−10.4375	10.2
1704721510.59075	0.7208	92.4375	−7.25	−9	13
1704721510.76959	0.8997	117.0625	−6.1875	−8.875	14
1704721510.89019	1.0203	145.5625	−4.9375	−8.8125	13.8
1704721511.01044	1.1405	158.5	−4.3125	−9.1875	11.8
1704721511.13109	1.2612	165.5625	−4.25	−9.125	9.2
1704721511.25108	1.3812	169.9375	−4.8125	−8.9375	7
1704721511.37038	1.5005	173.5625	−4.8125	−8.5625	4.8
1704721511.54824	1.6783	174.5	−5	−8.5625	3
1704721511.67071	1.8008	174.4375	−5.75	−9.0625	1.8
1704721511.79061	1.9207	175.375	−5.75	−9.625	1.2
1704721511.91064	2.0407	175.3125	−5.8125	−9.8125	0.6
1704721512.02992	2.1600	175.0625	−5.8125	−10	0.4
1704721512.15051	2.2806	174.5	−5.8125	−10.0625	0.4
1704721512.27115	2.4013	174.375	−5.875	−10.1875	0.4
1704721512.39028	2.5204	174.75	−6	−10.375	0
1704721512.51079	2.6409	174.875	−6.0625	−10.4375	0
1704721512.63037	2.76053	174.875	−6.1875	−10.5625	0
1704721512.75082	2.8809	174.875	−6.3125	−10.6875	0
1704721512.87001	3.0001	174.875	−6.4375	−10.8125	0

Rows are highlighted with different colors representing different stages of recorded Euler angles and RPM as the ball starts spinning. Orange-highlighted rows indicate values recorded while the ball has no rotation. Yellow-highlighted rows indicate RPM values averaged with 0 values before rotation began. Green-highlighted rows indicate RPM values that are averaged with non-zero previous values.

## Data Availability

All data supporting the findings of this study are contained within the article. Additional information related to this study are available from the corresponding author upon reasonable request.
