# Correction: Foo et al. Enhancing Tennis Practice: Sensor Fusion and Pose Estimation with a Smart Tennis Ball. *Sensors* 2024, *24*, 5306

**DOI:** 10.3390/s25061704

**Published:** 2025-03-10

**Authors:** Yu Kit Foo, Xi Li, Rami Ghannam

**Affiliations:** James Watt School of Engineering, University of Glasgow, Glasgow G12 8QQ, UK; fooyukit@outlook.com (Y.K.F.); 2756830l@student.gla.ac.uk (X.L.)

The authors would like to make the following corrections for their published paper [1].

Three new references, References [14,17,18], have been added:

14. 94 Fifty. 94 Fifty Smart Sensor Basketball. 2024. Available online: https://test-94fifty.myshopify.com (accessed on 23 August 2024).

17. CoachLabs Golf. Geni1 Smart Golf Ball. 2024. Available online: https://coachlabs.com/collections/geni1/products/gen-i1 (accessed on 23 August 2024).

18. Kookaburra Sport. Kookaburra Smart Ball. 2024. Available online: https://www.kookaburrasport.co.uk/cricket/team-kookaburra/innovation/?srsltid=AfmBOoojoKHWok740S_5swPei2_Olayswu6A4fm91AG7IOooD5Upvta5 (accessed on 23 August 2024).

The original references [16,17] have been replaced by the above references [17,18]. With this correction, the order of some references has been adjusted accordingly. 

The authors state that the scientific conclusions are unaffected. This correction was approved by the Academic Editor. The original publication has also been updated.
